# Prediction of Amyloid‐Beta PET Status with a Combination of Digital Clock and Recall, Digital Trail Making Test‐Part B, and Blood‐Based Biomarkers

**DOI:** 10.1002/alz70856_107699

**Published:** 2026-01-09

**Authors:** Ali Jannati, Karl Thompson, Claudio Toro‐Serey, Connor Higgins, David Bates, Alvaro Pascual‐Leone, Sean Tobyne

**Affiliations:** ^1^ Linus Health, Boston, MA, USA; ^2^ Harvard Medical School, Boston, MA, USA; ^3^ Hebrew SeniorLife, Boston, MA, USA

## Abstract

**Background:**

Early identification of patients who are likely to have abnormal brain amyloid‐beta (Aβ) levels is crucial for identifying patients with Alzheimer's disease (AD) and prioritizing suitable candidates for disease‐modifying treatments (DMTs). Therefore, there is an urgent need to develop an efficient, cost‐effective, and scalable method for early identification of brain Aβ+ status. This study evaluated the performance of ML‐enabled models combining the Digital Clock and Recall (DCR), digital Trail‐Making Test‐Part B (dTMT‐B), and various blood‐based biomarkers (BBMs) in predicting brain Aβ‐PET status.

**Method:**

930 participants (mean age 72.0±6.7; 56.8% female; 23% minorities) in the Bio‐Hermes‐001 study were classified as cognitively unimpaired, mild cognitive impairment, or probable Alzheimer's dementia, and 35.1% were Aβ+ on 18F‐florbetapir PET scan. Three‐class, cross‐validated ensemble models combined multimodal process‐based features of DCR and dTMT‐B performance including drawing metrics, temporal‐spatial features of stylus manipulation, speech and acoustic features, APOE status, and BBMs *p*‐tau217, Aβ42/40, *p*‐tau181. All models had <22% indeterminate cases.

**Result:**

For predicting Aβ‐PET status, the DCR+dTMT‐B+P‐tau217 model had an AUC=0.943 (NPV=0.946; PPV=0.871), and with APOE added, it achieved AUC=0.954 (NPV=0.963; PPV=0.907). The DCR+dTMT‐B+Aβ42/40 model had an AUC=0.929 (NPV=0.936; PPV=0.820), and with APOE added, it achieved AUC=0.948 (NPV=0.952; PPV=0.892). The DCR+dTMT‐B+P‐tau181 model had an AUC=0.911 (NPV=0.915; PPV=0.774), and with APOE added, it achieved AUC=0.941 (NPV=0.939; PPV=0.831). Addition of Aβ42/40 to the DCR+dTMT‐B+P‐tau181+APOE model resulted in an AUC=0.949 (NPV=0.960; PPV=0.883).

**Conclusion:**

A multimodal model based on DCR, dTMT‐B, ±APOE, and various BBMs had excellent performance in predicting Aβ‐PET status, at levels comparable to CSF biomarkers of AD. ML‐enabled digital cognitive assessments that leverage process‐based metrics, such as the DCR and dTMT‐B, enable cost‐effective integration into clinical workflows to identify patients who are most likely to have Aβ+ PET status, allowing for prioritizing the most suitable candidates for DMTs.

